# Reversible anomia and cerebral venous thrombosis: a case report and review of the literature

**DOI:** 10.1186/s13256-022-03264-3

**Published:** 2022-02-12

**Authors:** Biniyam A. Ayele, Riyad Ibrahim Abdella, Leilt Zewdie Wachamo

**Affiliations:** 1 Department of Neurology, College of Health Sciences, Addis Ababa University, Addis Ababa, Ethiopia; 2 School of Medicine, Welkite University, Wolkite, Ethiopia; 3 Ethiopian Public Health Institution, Addis Ababa, Ethiopia

**Keywords:** Cerebral venous thrombosis, Anomia, Focal neurologic deficit, Ethiopia

## Abstract

**Background:**

Cerebral venous thrombosis is a rare form of venous stroke with diverse clinical manifestations. Word-finding difficulty (anomia) is rarely reported in patients with cerebral venous thrombosis.

**Case presentation:**

We report a 30-year-old right-handed Ethiopian female patient, who presented with global headache associated with a new onset word-finding difficulty of 2 weeks duration. The headache was not responsive to over-the-counter medications. She reported blurring of vision and nausea. Two months previously, she gave birth to a dead fetus. On neurological assessment, the patient was fully conscious and oriented, with a Glasgow coma score of 15/15, and cranial nerves, motor, and sensory examinations were unremarkable. Examination of fundus showed grade 2 papilledema bilaterally. Language assessment showed normal fluency, compression, naming, reading, and repetition. Naming was assessed using a 60 second word generating test, which indicated anomia. Brain magnetic resonance imaging showed left temporoparietal ischemia, magnetic resonance venography showed thrombosis of the left transverse, sigmoid sinus, and corresponding cortical veins. She was started on warfarin 5 mg daily for 6 months and showed significant resolution of symptoms, including the anomia.

**Conclusion:**

The present case describes a young female patient with reversible anomia as a complication of cerebral venous thrombosis. The case also highlights the importance of timely diagnosis and treatment of cerebral venous thrombosis for a benign prognosis.

## Background

Cerebral venous thrombosis (CVT) is thrombosis of the cerebral veins and sinuses, which is often uncommon and misdiagnosed. CVT is a type of cerebrovascular disease that often presents with focal cerebral edema, venous cerebral infarction, seizures, and intracranial hypertension as its most prominent clinical features [1–3]. Of interest, in poor countries, there is an association with the puerperium, with no clear arguments, but probably related to factors such as inappropriate perinatal care, metabolic derangements, and infections associated to childbirth. Peripartum-associated CVT has been established to occur in 11.6 per 100,000 deliveries [4]. Rarely, CVT may present with language abnormality such as word-finding difficulty, also known as, anomia. Anomia is commonly associated with lesions involving the inferior temporal gyrus and adjacent parieto-occipital lobes of a dominant hemisphere [5, 6]. These particular regions of the brain are drained by the ipsilateral transverse sinus, cortical venues, and sigmoid sinuses [6–8]**.** The present case reports a patient with reversible anomia as a complication of cerebral venous thrombosis, highlighting the importance of timely diagnosis and treatment of CVT for a benign prognosis.

## Case presentation

**Fig. 1:**
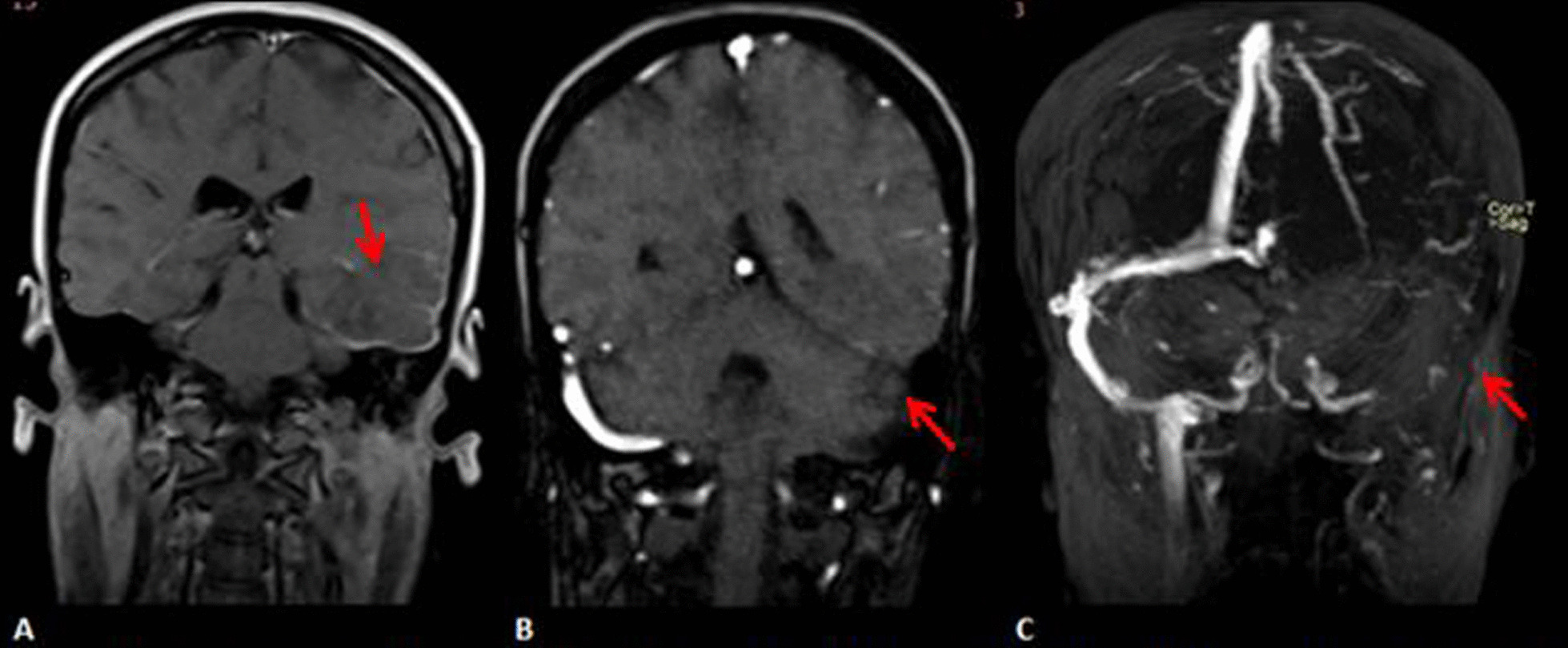
**A** Coronal T1 magnetic resonance imaging showing hypointense lesion in the left inferior parietal region (red arrow). Coronal (**B**) and 3-D (**C**) magnetic resonance venography showing filling defect of the left transverse and sigmoid sinuses along with corresponding cortical venues (red arrow)

We report a 30-year-old right-handed Ethiopian female patient, who presented with a global headache and word-finding difficulty of 2 weeks duration. The headache was not responsive to over-the-counter medications. She also reported blurring of vision and dizziness associated with the headache. Her past medical history was pertinent for giving birth to a dead fetus 2 months previously. Otherwise, no histories of previous stillbirth or miscarriage, oral contraceptive use, or of diabetes, hypertension, or cardiac disease were noted. On examination, her blood pressure (BP) was 130/70 mmHg, pulse rate (PR) 92 beats per minute, respiratory rate (RR) 13 breaths per minute, and temperature was 36.5 °C. On neurological assessment, the patient was fully conscious and oriented, with a Glasgow coma score (GCS) of 15/15, and cranial nerves, motor, and sensory examinations were unremarkable. Examination of fundus showed grade 2 papilledema bilaterally. Cognitive assessment of naming using a 60 second word generating test was significantly affected. She was able to name only one wild animal in 60 seconds. The other components of language were unaffected, including, fluency, comprehension, repetition, writing, and reading. Brain magnetic resonance imaging (MRI) showed inferior left temporoparietal ischemia (Fig. 1A), and brain magnetic resonance venography (MRV) showed thrombosis of the left transverse and sigmoid sinus along with corresponding cortical veins (Fig. 1B, C). Routine laboratory investigations were unremarkable. Due to the patient’s financial problems, detailed thrombophilic workups such as factor V, protein C, and protein S were not possible.

Once the diagnosis of CVT was confirmed, the patient was admitted to the medical ward and started on bridging heparin of 17,500 IU subcutaneously twice a day for 4 days, then warfarin 5 mg daily was initiated. She was discharged home with oral anticoagulation after being on the ward for 7 days and her symptoms had started to improve. On follow-up evaluation (after 1 month), her headache has disappeared and her anomia showed significant improvement, evidenced by significant improvement at the follow-up assessment using the 60 second word generating test. On her second follow-up (at 6 months), the patient’s clinical symptoms and signs were fully resolved. However, we could not get a follow up MRI and MRV because of the patient’s financial problems. We discontinued the oral anticoagulation after 6 months of treatment, considering transient CVT risk factors such as being postpartum.

## Discussion and conclusion

Cerebral venous thrombosis is a rare and potentially fatal type of stroke, with estimated incidence of 5 per 1 million [1]. CVT has preponderance in females and a younger age group. If not treated properly, 4.3% of patients die during the acute phase of CVT [4, 9]**.** There are many risk factors of CVT including hereditary thrombophilia, acquired hypercoagulable, and hyperviscosity states such as hyperhomocysteinemia, antiphospholipid antibody syndrome, Behcet’s disease, and hematological disorders. Furthermore, pregnancy, puerperium, and oral contraceptive usage are also common culprits for CVT development [10, 11]**.** We present a case of a young woman with reversible anomia associated with ischemia of the left temporoparietal region and thrombosis of the left transverse and sigmoid sinuses following 6 months of anticoagulation therapy. Headache is the most common presenting symptom of CVT and is reported in 80–90% of cases [1, 3, 12, 13]. The present case reported word-finding difficulty associated with headache. We have searched Google scholar and PubMed databases for published cases of “isolated anomia associated with cerebral venous thrombosis” and found two cases, which are summarized in Table 1 [14, 15].

**Table 1:** Cases of cerebral venous thrombosis presenting with reversible anomia, including the present case

	Authors	Age/sex	Presenting symptoms	Risk factors	Affected cerebral sinuses	Affected cerebral region	Treatment	Outcome
1	Kuan *et al*. (2014)	52/F	Headache anomia Nausea Vomiting	Protein C and protein C deficiency	Left transverse	Left parietotemporal	Heparin followed by warfarin	Improved
2	Sarma *et al*. (2004)	23/F	Headache anomia Nausea Vomiting	Oral contraceptive	Superior, inferior, vein of Galen, and straight sinuses	Bilateral parietotemporal, basal ganglia, and thalami	Heparin followed by warfarin	Improved
3	Ayele *et al*. (2021)	30/F	Headache anomia Blurred vision	Postpartum	Left transverse and sigmoid sinuses along with the corresponding cortical veins	Left parietotemporal	Heparin followed by warfarin	Improved

A cerebral venous thrombosis is more common in female patients and often affects young women, with a mean age of 30–40 years [16]. This is consistent with the demography of the present case. However, Kuan *et al*. 2014 [15] reported the case of a 52-year-old woman with CVT complicating with anomia. Several risk factors were associated with CVT; in this case, we have attributed the cerebral sinus thrombosis to transient risk factors such as the postpartum period. This is contrary to the previously reported cases, in which CVT was attributed to protein C and protein S deficiency and oral contraceptive usage (Table 1). The caveat to the present case is the lack of extensive thrombophilic workup of the patient due to financial problems. In the present case, the presence of headache, nausea, vomiting, and bilateral papilledema indicated increased intracranial pressure (ICP), which occurs secondary to obstruction of cerebrospinal fluid (CSF) absorption, which is consistent with prior studies [9, 13, 17]. Rarely, patients with CVT may present with word-finding difficulty when the parietotemporal region of the dominant hemisphere is affected as a result of thrombosis of transverse, superior, and sigmoid sinuses [14, 15].

Naming is a complex language function that utilizes input from multiple cortical regions [8]. However, anomia is commonly associated with lesions involving the inferior temporal gyrus and adjacent parieto-occipital lobes of a dominant hemisphere [5, 6]. These particular regions of the brain are drained by the ipsilateral transverse sinus, cortical venues, and sigmoid sinuses [6–8]**.** This is in congruence with the present case and previously reported similar cases (Table 1). The present case was treated with anticoagulation for 6 months and showed significant clinical improvement including reversal of the anomia. This is consistent with the previously reported cases, which showed benign outcomes after 3–6 months of anticoagulation treatment [14, 15].

In summary, the present case describes a young female patient with reversible anomia as a complication of cerebral venous thrombosis. The case also highlights the importance of timely diagnosis and treatment of CVT for a benign prognosis.

## Data Availability

All data sets on which the conclusions of the case report based, are to be available as spreadsheets and are available from the corresponding author on reasonable request from the editors.

## Abbreviations

CVT: Cerebral venous thrombosis
DWI: Diffusion-weighted imaging
MRI: Magnetic resonance imaging
MRV: Magnetic resonance venography
